# Annotations

**Published:** 1909-10-30

**Authors:** 


### ANNOTATIONS.

### Milton's Blindness.

In a most lucid paper contributed to the Oxford Ophthalmological Congress this year, Professor Dufour, of Lausanne, gives an account communicated by the poet himself to a friend in Paris of the exact symptoms and mode of onset of Milton's blindness. It was in 1644 that the visual field of the left eye was encroached upon by a shadow which extended gradually to complete amblyopia. Haloes were seen round flames, and motionless objects appeared to float about, while a cloud of steam seemed to hang around his forehead. The right eye was then involved in a somewhat similar way, but the pall of darkness was never absolutely black: sensations of faint light, compared by Milton with early dawn, still remained in 1654, the date of the letter in question, written for the benefit of a Paris oculist from whom some advice was expected. In the right eye, it should be mentioned, the upper half of the visual field was the first to be lost. These symptoms, says Professor Dufour, point conclusively to detachment of the retina. No mention is made of any shock or injury in association with the blindness; but from the age of twelve Milton admits having worked daily up to midnight. This devotion to literary study is at once evidence of extreme short sight, and a plausible cause for its progressive course and its ending in detachment of the retina. The author concludes with the humiliating truth that, though we might succeed better than our forefathers in diagnosing the complaint, we should be just as powerless to cure it.

### Yellow Fever in Rio de Janeiro.

The wonderful results obtained in Havana in the prophylaxis of yellow fever by the splendid work of Gorgas and his assistants during the occupation of that city by the United States Government are well known to medical readers. A similar satisfactory result has now been brought about in Rio de Janeiro, which in days gone by rivalled the Cuban capital as the worst yellow fever depôt of the world. In 1903 the Public Health Service of Rio organised a bureau of yellow fever prophylaxis under the direction of Dr. Carneiro de Mandonça, who unfortunately died before his labours brought forth fruit. However, backed by a grant from Congress, a large force of skilled and unskilled workers was organised, and the entire town was carefully inspected after the methods of Gorgas in Havana. What an immense labour this has entailed may be realised when it is mentioned that the city contains nearly a million inhabitants, and over 82,000 houses spread over an area of 430 square miles of hilly country. The most approved system of prophylaxis was carried out, and in 1905 there were but 289 deaths from this disease. The next year the number fell to 42, in 1907 it was 39, in 1908 to four. So far there have been no deaths at all this year, and as the usual season for the outbreak is already over, it may be confidently hoped that there will be none. This extremely satisfactory state of things is greatly to the credit of the bureau, and its chief, Dr. Cruz, who read a paper on this subject in August before a medical congress at Rio, is to be warmly congratulated on the success achieved. The story shows that Gorgas' work in Havana constitutes a claim on the world's gratitude such as few even of the medical profession are able to establish.

### The Cause of Epilepsy.

In the fourteenth annual report of the Lanark District Asylum appears the medical statistical and general summary furnished by Dr. Neil T. Kerr, the Medical Superintendent, who appears to have devoted much spare time to investigations into the ætiology and pathology of epilepsy. After paying considerable attention to the routine microscopic and cytological examination of the blood of epileptic patients, he states that pure epilepsy (so called) is not accompanied by any change in the number or character of the white blood corpuscles or of any other of the blood elements. There are, however, certain cases where there is a definite leucocytosis corresponding to each increase in the number of seizures. This is usually accompanied by an increase in the number of eosinophiles. These conditions, Dr. Kerr thinks, may be due to some extraneous factor (perhaps that which causes the mental symptoms), but it is tempting to include a toxic type in our classification of epilepsy. With regard to this toxin, he is of opinion that auto-intoxication from constipation is without doubt an exciting factor. Dr. Kerr concludes that, to give any opinion on the pathology of these conditions would be rash at this time of controversy, but no doubt this branch will be elucidated in the near future, and much of the "kudos" should go to those observers in the wards who keep accurate record of the paroxysmal and interparoxysmal periods of epilepsy.

### Doctors and Midwives.

An interesting inquest held before Dr. Waldo a fortnight ago draws attention once more to certain anomalies in the law relating to midwives and the relations between doctors and midwives generally. The deceased in this case was apparently delivered by a woman whose name had been struck off the midwives' roll, and who had twice unsuccessfully attempted to become rehabilitated, and who had called in a local medical man after the birth of the child. The evidence showed that the child was still-born, with a developed mental defect in the left side of the diaphragm. Dr. Waldo, in instructing the jury, drew attention to the fact that under the new Midwives Act only qualified women whose names appeared on the roll are allowed to practise in England, while, as far as the medical profession is concerned, every untrained, uneducated, and dangerous quack is given a liberty to experiment upon the public. At another inquest before the same Coroner the question of fees to doctors called in by midwives in urgent cases arose. In this case the medical man, as so often happens, had given his services free, and had been put to considerable expense of time and trouble. Such cases, it appears to us, should be referred to the Poor Law officers, but it is quite obvious that this course often entails considerable loss of time, and therefore in these urgent cases danger to life, since the relieving officer is not always easily to be found.

